# Effectiveness of ultrasonography-guided periforaminal oxygen-ozone therapy in chronic low back pain

**DOI:** 10.55730/1300-0144.6015

**Published:** 2025-04-24

**Authors:** Esra ÜLGEN KIRATLIOĞLU, Kutay TEZEL, Eda GÜRÇAY

**Affiliations:** Department of Physical Medicine and Rehabilitation, Gaziler Physical Medicine and Rehabilitation Education and Research Hospital, Health Sciences University, Ankara, Turkiye

**Keywords:** Ozone, epidural injections, ultrasound guidance, low back pain

## Abstract

**Background/aim:**

Treatment of chronic low back pain (LBP) includes various minimally invasive image-guided interventional techniques. The aim of this study, which was planned based on the idea that periforaminal oxygen-ozone (O2-O3) gas application with ultrasonography (USG) could be an alternative treatment option, was to compare the therapeutic efficacy of USG-guided periforaminal ozone infiltration (PFOI) or transforaminal epidural steroid injection (TFESI) in patients with chronic radicular LBP on pain, disability, and quality of life (QoL).

**Materials and methods:**

A total of 50 patients were randomly divided into 2 groups of 25 patients each underwent PFOI by USG or TFESI via fluoroscopy. Pain intensity was assessed with visual analogue score (VAS), disability with Oswestry disability index (ODI), and QoL with short form-36 (SF-36), before treatment, at the 2nd week, 1st month, and 2nd month, after treatment.

**Results:**

Intra-group comparisons showed significant improvements in LBP VAS, radicular pain VAS, and ODI after treatment for both groups. The reduction in LBP VAS was found to be higher in the PFOI group at week 2 (p = 0.010) and month 1 (p = 0.037), and the improvement in ODI scores was significantly better in the PFOI group at week 2 (p = 0.017) compared to the TFESI group.

**Conclusion:**

Our findings have shown that PFOI and TFESI have positive effects on chronic radicular LBP, disability, and QoL parameters. Since ultrasound-guided O2-O3 injections were found to be more effective than epidural steroid injections, particularly in the early period, it was suggested that O2-O3 therapy may be considered in the preoperative treatment algorithm of patients with chronic radicular LBP.

## 1. Introduction

Low back pain (LBP) with radicular pain, a common cause of activity limitation, is often caused by lumbar disc herniation [1]. Compression or neuroinflammation at the spinal nerve root or dorsal root ganglion induces inflammatory reactions that lead to radicular pain in the lower extremity [2]. The prevalence in the general population has been reported between 9.9% and 25% [3]. Patients with persistent pain or with neurological deficits need surgical interventions. Otherwise, consequences such as disability, loss of productivity, and deterioration in quality of life become inevitable [4].

Conservative treatments include a healthy lifestyle, modification of activities, oral analgesics, nonsteroidal anti-inflammatory drugs, neuropathic agents, manual therapy, posture and motor control exercises, and physical therapy modalities [5]. Although microsurgical methods are preferred and applied more frequently at the end of the first year after surgery, pain reduction is limited to 67%–76% [6], and failed back surgery syndrome develops in approximately 25% [7] of cases.

Many new minimally invasive image-guided interventional techniques have been developed to prevent long-term exposure to systemic drugs and to reduce the need for surgery in patients who do not benefit from standard conservative treatments. Therefore, epidural steroid and local anesthetics can be considered one of the most common non-surgical treatment methods for lumbar disc herniation [8,9]. The transforaminal technique is preferred as the most effective route because it provides drug access directly to the area where the disc herniation compresses the inflamed nerve root [5,10–14].

Oxygen-ozone gas (O2-O3) has been proven to be effective in reducing pain in many musculoskeletal disorders, including LBP, neck pain, cervical/lumbar disc herniation, failed back surgery syndrome, and degenerative spine disease [15]. O2-O3 has antiinflammatory and antioxidant mechanisms. Similar to steroids, it inhibits inflammatory cytokines, reduces prostaglandin synthesis, ischemia, and edema in inflamed nerve roots, and additionally increases tissue oxygenation by increasing microcirculation and neoangiogenesis [16]. In recent years, studies have been conducted in which O2-O3 was applied with different techniques such as direct intradiscal approach by radiological guidance (CT, fluoroscopy) or indirect blind paravertebral approach in spinal pain [16–18]. Different radiological methods have been used in transforaminal/periforaminal applications targeting the region where the disc material compresses the nerve root, and ultrasonography (USG) has become safely used method in many clinical practice due to its high accuracy and precision it provides [19–21]. To the best of our knowledge, there is no current study in the literature comparing transforaminal epidural steroid injection (TFESI) with PFOI. Therefore, periforaminal O2-O3 gas application with USG can be offered as an alternative option. The aim of this study was to compare the therapeutic efficacy of USG-guided PFOI versus TFESI in patients with chronic radicular LBP on pain, disability, and quality of life (QoL).

## 2. Materials and methods

### 2.1. Patients’ selection

This prospective randomized controlled study included 50 patients aged 20–75 years suffering from chronic LBP with radicular pain, who were admitted to Gaziler Physical Medicine and Rehabilitation Training and Research Hospital. Patients had a pain level of ≥4 on the visual analog scale (VAS) and evidence of disc herniation on magnetic resonance imaging (MRI). Chronic LBP is defined as pain occurring posteriorly in the region between the lower rib margin and the proximal thigh for more than 3 months. Radicular pain is defined as pain radiating down the posterior or lateral part of the leg beyond the knee, with positive physical findings on nerve tension tests (i.e. straight leg raise, Lasegue, or Bragard tests).

All patients completed at least 4 weeks of conservative treatment with analgesics and physical therapy modalities without clinical improvement. Exclusion criteria were previous spinal surgery/stenosis, spondylolysis, spondylolisthesis, cauda equina syndrome/progressive neurological deficit, neurogenic bladder/bowel syndrome, peripheral neuropathy, neurodegenerative diseases, vascular disease/claudication, local infection at the injection site, bleeding diathesis, uncontrolled diabetes mellitus, hyperthyroidism, hypertension, history of malignancy, pregnancy, body mass index (BMI) > 40, and a history of allergy to the injection solutions used in this study or glucose-6-phosphate dehydrogenase deficiency (favism), in which O2-O3 treatment was contraindicated.

Patients were randomly assigned to the study group (PFOI group, n = 25) or control group (TFESI group, n = 25) in a 1:1 ratio using a computer-generated allocation array.

### 2.2. Ethical approval

This study was conducted after obtaining approval from the local ethics committee with the identification number E-95961207-604.01.01-1588. The study protocol was registered in the clinicaltrials.gov database (NCT05586633). All included patients provided written informed consent for the treatment and use of their clinical data for scientific purposes. All the procedures were carried out in accordance with the 1975 Helsinki Declaration, as revised in 2000.

### 2.3. Outcome evaluation

At presentation, each eligible patient underwent a complete physical examination and blood biochemical and hematological parameters, in some cases an electrophysiological study, and other radiological tests were performed when necessary.

Pain intensity was measured using a standard 10-cm visual analog scale (VAS), with 0 indicating “no pain” and 10 indicating “the worst pain ever experienced” [22].

The Turkish version of the Oswestry disability index (ODI) is a patient-filled questionnaire used to assess functional impairment due to pain [23]. This index, which evaluates the impact of LBP on activities of daily living, consists of 10 questions and is scored from 0 to 5. The score is calculated as a percentage by dividing the patient’s total score by 50, the highest score that can be obtained from the test. Higher scores correspond to a higher level of disability.

The QoL was evaluated with Short Form-36 (SF-36), which consists of 8 sections: physical functioning, physical role limitation, emotional role limitation, energy/fatigue, emotional well-being, social functioning, pain, and general health [24]. Each component of the SF-36 provides a total score ranging from 0 (worst) to 100 (best health status).

To define the effectiveness of the procedures, a 2-month follow-up was performed. Outcome measurements were recorded before treatment (T0), at the 2^nd^ week, (T1) 1^st^ month (T2), and 2^nd^ month (T3), after treatment. All of the assessment parameters were self-reported by the patients without the influence of the researchers.

### 2.4. Intervention

The treatment level was determined based on the patient’s reported symptoms, MRI findings, and neurological examination results by the physiatrist.

#### 2.4.1.Transforaminal epidural steroid injection (TFESI)

The procedures were always performed on outpatients by the same physiatrist (K.T. has 20 years of experience in spinal interventions with fluoroscopy). Prior to the procedure, peripheral venous access was established in each patient to manage any potential complications or side effects. Upon arrival in the operating room, vital signs (blood pressure, pulse oximetry, and electrocardiography) were monitored as standard.

**Figure 1:**
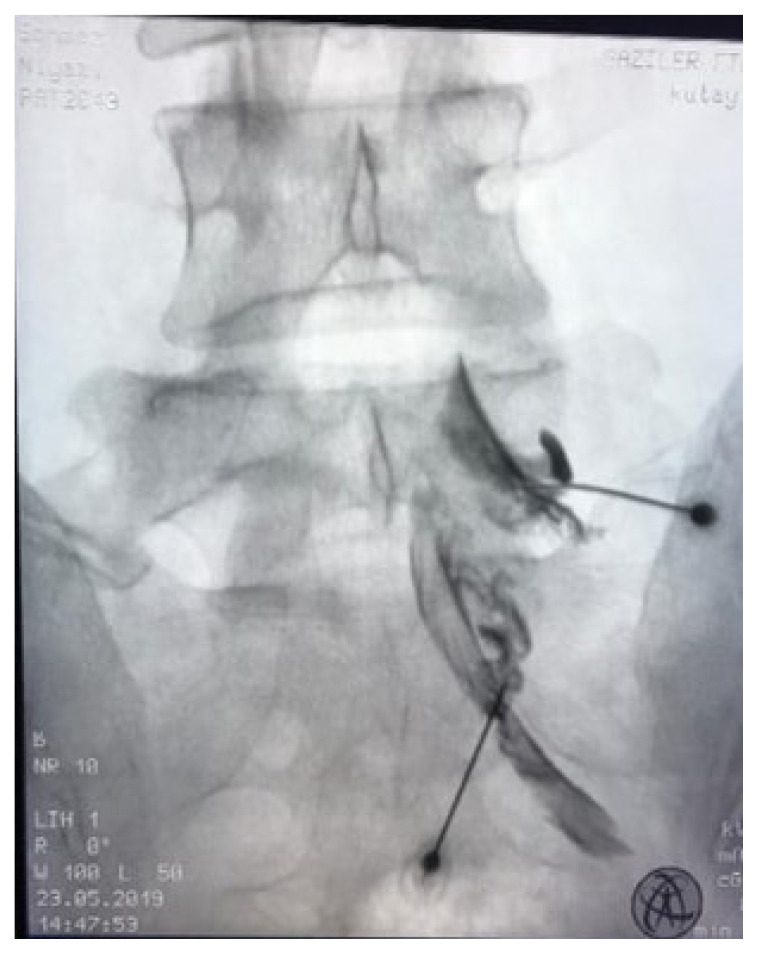
In the fluoroscopic lumbar anteroposterior image, the spread of the contrast material applied to the L5 and S1 nerve roots is observed.

The patients were placed in a prone position on the fluoroscopy procedure table, and a thin pillow was placed under the abdomen to flatten the lumbar lordosis. Since no premedication or anesthesia was administered, patients remained awake during the procedure. The injection site was disinfected using ethyl chloride spray for aseptic procedure. The operator determined the target level on the posteroanterior view of the lumbar spine with C-arm fluoroscopy. After determining the target vertebra, the C-arm fluoroscopy was adjusted in the cephalocaudal direction so that the x-ray beams passed parallel to the end plates and a “square vertebra” image was obtained. At this point, the fluoroscope was rotated obliquely (30–45°) to the affected side, and a “Scottish dog” sign was observed on the x-ray screen. The skin was anesthetized by subcutaneous injection of 2 mL 1% lidocaine hydrochloride. A Quincke type 21-gauge 90-mm (3.5 inch) spinal needle was advanced into the “safe triangle” region formed by the pedicle, vertebral body, and the existing nerve root [25]. On the lateral view, the needle tip was inserted under the pedicle at the ventral aspect of the intervertebral foramen. Once the target point was reached, 1–2 mL of contrast was injected and an image was taken to ensure that the needle was not in a vascular location. After the contrast material was observed to spread in the epidural space, 2 mL of 2% lidocaine HCL and 8 mg/2 mL dexamethasone were injected, and the procedure was terminated (Figure 1).

#### 2.4.2.Periforaminal O2-O3 injection (PFOI)

**Figure 2:**
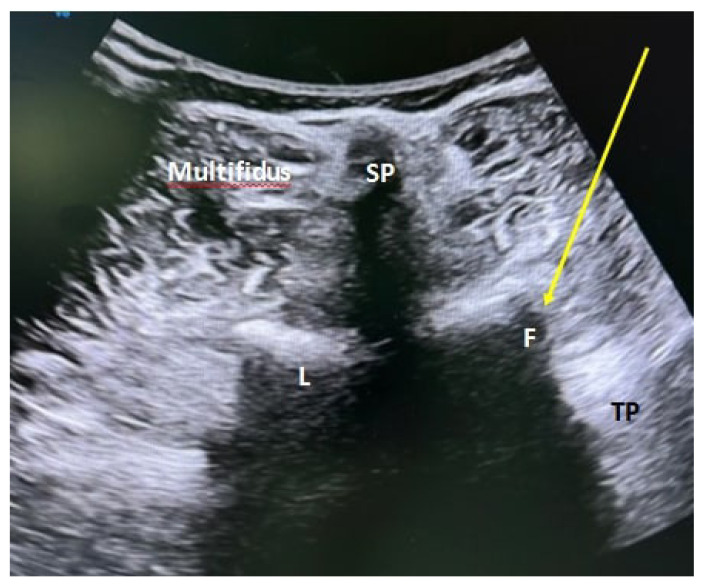
An axial view was obtained when the probe was rotated 90° over the L5 spinous process. The spinal needle was inserted into the skin at an angle of approximately 45° and advanced from lateral to medial using an in-plane approach targeting the facet joint. SP, spinous process; F, facet joint; L, lamina; TP, transverse process; yellow arrow, trajectory of spinal needle.

**Figure 3:**
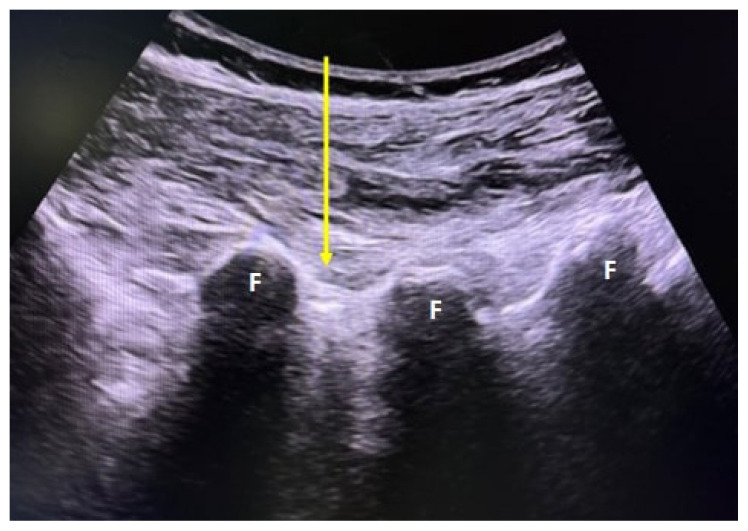
The probe was rotated 90˚ in the axial position over the L5 spinous process and when the needle tip was seen, it was gently advanced between two consecutive facet joints. F, facet joint; yellow arrow, trajectory of spinal needle.

Patients undergoing the PFOI procedure were positioned on the examination table in the outpatient intervention room in the prone position. Sonographic evaluations were performed with a 5–12 MHz curvilinear transducer (Logice portable; GE Healthcare, China) by the same physiatrist who had 5 years of experience in USG-guided lumbar injections. In order not to reduce the effectiveness of the O2-O3 gas, the preparation of O2-O3 in the device (Evozone, GmbH D-72762 Reutilingen, Germany) was performed simultaneously with sonographic assessment. The injection site was prepared for aseptic processing in accordance with sterile technical conditions. Initially, the probe was placed on the spinous processes in the sagittal plane to visualize the L5 spinous process and the median crescent of the sacrum. The desired spinal level was determined by counting the spinous processes as the probe was advanced cephalad. Once the planned injection level was reached, the probe was rotated 90 degrees over the spinous process to obtain a transverse view (Figure 2). The skin was anesthetized with a subcutaneous injection of 2 mL of 1% lidocaine hydrochloride. A Quincke type 21-gauge 90-mm (3.5 inch) spinal needle was inserted into the skin at an angle of approximately 45° and advanced from lateral to medial using an in-plane approach. In this position, the needle was advanced to the lateral side of the lamina, then the probe was rotated 90°, allowing visualization of the needle tip in the parasagittal plane in the middle of two consecutive facet joints (Figure 3). The needle tip was then directed slightly outward and advanced a few millimeters deeper without any resistance. Following negative aspiration, O2-O3 gas was injected into the targeted nerve root.

The treatment regimen consisted of a total of 4 sessions twice a week. In the first week, 5 mL of O2-O3 at a concentration of 10 μg/mL was applied slowly and unilaterally to each affected level, while in the second week, 5 mL of O2-O3 at a concentration of 15 μg/mL was applied to the same levels. Although there is no standardized treatment protocol in terms of O2-O3 gas concentration, volume, and frequency of application among previous study protocols, we modified treatment protocol within the limits declared in the Madrid Declaration [26].

In both groups, patients were observed for complications and adverse effects for at least 30 minutes after the procedure and were discharged after a final examination.

### 2.5. Statistical analysis

Data were analyzed through StataMP13 (StataCorp. Stata Statistical software: Release 13). The conformity of continuous variables to a normal distribution was examined using the Shapiro–Wilk test. Descriptive statistics were shown as mean ± standard deviation (SD) or median (minimum–maximum) for continuous variables, and as frequency (n) and percentage (%) for categorical variables. An independent simple t-test was used for parametric data and Mann-Whitney U test was used for non-parametric data in pairwise group comparisons. Comparisons for categorical variables were evaluated with chi-squared and Fisher’s exact tests. Wilcoxon test for 2 groups in continuous variables, and the Friedman test for multiple groups. A p value of <0.05 was considered statistically significant.

## 3. Results

A total of 120 patients with chronic LBP were screened for the study, and 50 patients who met the criteria were included. Demographic and clinical data of the patients (symptom duration, side, level and number of treatments, LBP and radicular pain levels, disability, and quality of life scores) were collected. The patients were homogeneous in terms of variables except for the SF-36 “general health status” subscale (p = 0.016) (Table 1). The most frequently treated spinal levels were L5, L5+S1, and S1, respectively.

Intra-group comparisons showed significant improvements in LBP VAS, radicular pain VAS, and ODI after treatment for both groups. Detailed analysis revealed that this difference was mainly due to the 2^nd^ week and 1^st^ month results. The reduction in LBP VAS was found to be higher in the PFOI group at week 2 (p = 0.010) and month 1 (p = 0.037), and the improvement in ODI scores was significantly better in the PFOI group at week 2 (p = 0.017) compared to the TFESI group (Table 2). For SF-36 comparisons, significant improvements were obtained in all subscales at some follow-up visits after treatment in both groups, except for the “energy” subscale for the TFESI group. “Physical functioning”, “emotional role limitation”, “energy/fatigue”, “pain”, and “general health” subscales showed more significant improvement at some follow-up time points after treatment in favor of the PFOI group (Table 2).

## 4. Discussion

Image-guided minimally invasive interventional techniques are important treatment options for patients suffering from chronic radicular LBP. Epidural steroid injections are currently preferred methods with increasing popularity due to their reliable and effective results. In recent years, ozone therapy has been applied through different routes in LBP patients, and the periforaminal technique has been addressed in a limited number of studies [27,31]. Therefore, in our study, TFESI, a remarkable treatment choice in clinical practice, was compared with PFOI and their effectiveness in terms of pain, disability, and QoL was evaluated. This study was planned based on the hypothesis that O2-O3 therapy may be more effective than epidural injections when delivered accurately and reliably to the pathological area around the disc and the impinged neural root under ultrasound guidance.

Our data showed significant improvements in LBP, radicular pain, disability, and QoL in the short and intermediate periods for both treatment modalities, consistent with previous studies [28,32,33]. The decrease in LBP was found to be higher in the PFOI group compared to the TFESI group, especially in the early periods. Igarashi et al., referring to the increased inflammatory cytokine levels in the facet joint tissues of patients with lumbar disc herniation, emphasized that the facet joints are responsible for part of the radicular pain [34]. From this perspective, it can be considered that the periforaminal intervention performed under USG guidance is technically closer to the facet joint. Additionally, the rapid spread of O2-O3 in the tissues, due to its gas structure, provides better penetration into the facet joints. The reason for the better scores in the ODI and several SF-36 subscales in the PFOI group may be interpreted as a reflection of reduced pain in LBP.

Different techniques and treatment protocols for O2-O3 in patients with LBP have been discussed in the literature [17,35]. Intradiscal approach, defined as the direct method, has both chemonucleolysis and mechanical effect to reduce/disappear the herniated material. Clinical success rates have been reported as 70%–80% [17]. The level of evidence for intradiscal O2-O3 therapy in LBP due to disc herniation is II-3 and for paravertebral and periforaminal O2-O3 therapy is II-1. The recommendation for intradiscal O2-O3 therapy is 1C and for O2-O3 applied to the paravertebral muscles or periforaminal is 1B [17,27]. On the other hand, the disadvantages of the intradiscal procedure are that it is performed under fluoroscopy or CT guidance and sedation, requires antibiotic prophylaxis and a strong infrastructure (equipment, different health professionals) [26], and it is recommended to avoid heavy activities for at least two weeks [27]. Furthermore, O2-O3 therapy has case-based reported complications such as intrathecal puncture, sensory retention after percutaneous intradiscal infiltration, subcutaneous hematoma at the injection site, and pyogenic spondylodiscitis [36]. It is important to emphasize that all of these adverse effects should be considered as side effects related to the delivery technique and not as side effects related to O2-O3 itself.

Paravertebral approach is defined as an indirect method that injects O2-O3 into the tender points corresponding to the herniated disc [37]. It can be performed in outpatient clinics without the need for imaging methods or premedication. However, it can lead to serious side effects and complications (acute muscle/visceral pain, fatal air embolism, burning or heaviness sensation) due to the large volumes required [36].

Morselli et al. compared O2-O3 therapy in two groups, one with a 10 mL (20 mcg/mL) via manual palpation (over the anatomical landmark) paravertebrally, and the other with a reduced dose of 5 mL (20 mcg/mL) by ultrasound, 10 times per week for both groups [38]. They reported that both procedures were equally effective in reducing pain and that the smaller volumes applied with ultrasound were safer and more comfortable. Bonetti et al. compared the clinical outcomes at short, intermediate, and long-term follow-up in patients who underwent single CT-guided intraforaminal (3 mL, 25 μg/mL) ozone gas infiltration or periradicular 2 mL steroid (80 mg, methylprednisolone) infiltration. They concluded that both steroid and O2-O3 therapy are highly effective in relieving acute and chronic LBP, and O2-O3 can be applied as a first-line treatment instead of epidural steroids [29]. Ryska et al. compared the efficacy of transforaminal single injection of 4–5 mL of an O2-O3 mixture (24 g/mL) therapy to that of TFESI and pulsed Radiofrequency (RF) in patients with chronic unilateral radicular syndrome [28]. The authors reported, although the TFESI group showed the early largest reduction in the VAS score among the groups, there was a decrease in pain levels and functional improvement (ODI scores) in all three groups with respect to the baseline at all follow-up time points, with no significant differences between groups. Sconza et al. conducted a study including patients with chronic LBP associated with sciatica and administered USG-guided periforaminal 10 mL O2-O3 of 20 μg/mL, 10 times on each side (total 20 mL) [39]. Numerical rating scale and ODI scores showed a significant improvement from baseline to the first evaluation performed 1 month after completing the treatment. However, ODI and SF-12 scores revealed stable results compared to the initial evaluation. However, there is still no clear consensus in the literature on the application site, dose, and repetition number specifically for image-guided periforaminal O2-O3 application. Since it was documented that adhesions in the roots due to repeated O2-O3 applications can complicate the surgical procedure, it has been recommended that conventional O2-O3 protocols be reviewed in this regard [17]. In our study, periforaminal O2-O3 was applied at a concentration of 10–15 μg/mL, which was compatible with guideline limits [26]. The same guideline also recommended that injections be administered twice a week for the first two weeks and once a week for 6 weeks for reinforcement after clinical improvement is observed. Based on our clinical experience of significant improvement after 4 injections (5 cc) in the first 2 weeks, we planned our study by limiting the number of injections to 4 in order to reduce the risk of serious complications and to increase patient compliance with the treatment due to repeated injections. Therefore, we modified the Madrid protocol by performing fewer injections at minimal doses under USG guidance, providing equivalent improvement that achieved with epidural steroid injections under fluoroscopic guidance.

The main limitations of our study included the relatively small population size and the 2-month follow-up period, which prevented the evaluation of long-term effects of treatment. Although the study was designed randomly, blinding was not applied to the investigators is an additional limitation. Advantages of the study included the homogeneity of the groups and the target-specific application of injections with image guidance.

Our findings have shown that periforaminal O2-O3 and transforaminal steroid injections have positive effects on LBP, disability, and quality of life parameters. Since ultrasound-guided O2-O3 injections were noted to be more effective than epidural steroid infiltration, particularly in the early period, O2-O3 therapy may be considered as a treatment option in patients with chronic radicular LBP before possible surgery. Studies with larger populations and longer term follow-up are necessarily needed to confirm this preliminary study.

## Figures and Tables

**Table 1:** Comparison of demographic and clinical variables of the patients.

Variables		PFOI group n = 25	TFESI group n = 25	p
**Age** (year), mean ± SD		47.76 ± 12.58	49.96 ± 11.73	0.525^b^
**Sex**, n (%)	Male	13 (52)	11 (44)	0.571^a^
	Female	12 (48)	14 (56)	
**BMI** (kg/m^2^), mean ± SD		27.69 ± 3.78	27.67 ± 3.20	0.988^b^
**Education level**, n (%)	Illiterate	0 (0)	0 (0)	0.190^a^
	Primary school	9 (36)	6 (24)	
	High school	5 (20)	11 (44)	
	University	11 (44)	8 (32)	
**Duration of symptom** (month), mean ± SD		23.20 ± 24.90	21.64 ± 20.93	0.654^c^
**Side of treatment**, n (%)	Right	12 (48)	12 (48)	1.000^a^
	Left	13 (52)	13 (52)	
**Level of treatment**, n (%)	L2+L3	0 (0)	1 (4)	0.790^a^
	L3+L4	1 (4)	1 (4)	
	L4	3 (12)	1 (4)	
	L4+L5	1 (4)	1 (4)	
	L5	9 (36)	9 (36)	
	L5+S1	4 (16)	7 (28)	
	S1	7 (28)	5 (20)	
**Number of treatment**, n (%)	1	20 (80)	16 (64)	0.208^a^
	2	5 (20)	9 (36)	
**LBP VAS**, mean ± SD		7.20 ± 0.29	7.96 ± 1.59	0.075^b^
**RP VAS**, mean ± SD		7.60 ± 1.32	7.80 ± 1.50	0.619^b^
**ODI**, mean ± SD		37.74 ± 13.76	47.02 ± 18.63	0.050^b^
**SF-36**, mean ± SD	Physical functioning	49.00 ± 16.52	45.45 ± 21.87	0.530^b^
	Physical role limitation	20.00 ± 28.87	18.18 ± 25.80	0.906^c^
	Emotional role limitation	49.32 ± 42.08	28.80 ± 38.90	0.090^b^
	Energy/Fatigue	49.60 ± 17.32	44.09 ± 16.01	0.265^b^
	Emotional well-being	65.28 ± 17.62	58.91 ± 16.62	0.210^b^
	Social functioning	55.50 ± 25.54	48.34 ± 21.29	0.306^b^
	Pain	29.50 ± 13.19	22.05 ± 13.49	0.062^b^
	General health	55.20 ± 18.96	42.27 ± 16.09	**0.016** b

PFOI, Periforaminal Ozone Injection; TFESI: Transforaminal Epidural Steroid Injection;

BMI, Body Mass Index; LBP, Low Back Pain; RP, Radicular Pain; VAS, Visual Analogue Scale; ODI, Oswestry Disability Index; SF-36, Short Form-36

SD, Standard Deviation

a , Chi-squared test;

b , T-test;

c , Mann-Whitney U test

**Table 2:** Comparison of LBP VAS, RP VAS, ODI, and SF-36 parameters within and between groups before and after treatment periods.

Parameters		PFOI group n = 25, mean ± SD		TFESI group n = 25, mean ± SD		p
**LBP VAS**	T0	7.20 ± 0.29		7.96 ± 1.59		0.075^a^
	T1	4.84 ± 2.10	**0.000**^d^, p1	6.36 ± 1.93	**0.000**^d^ p1	**0.010** a
	T2	3.68 ± 1.70	**0.001**^d^, p2	4.92 ± 2.34	**0.000**^d^ p2	**0.037** a
	T3	3.44 ± 2.36	0.216^d^, p3	4.72 ± 2.59	0.154^d^ p3	0.065^b^
	**p**	**0.000** c		**0.000** c		
**RP VAS**	T0	7.60 ± 1.32		7.80 ± 1.50		0.619^a^
	T1	5.00 ± 2.02	**0.000**^d^, p1	5.72 ± 2.21	**0.000**^d^ p1	0.235^a^
	T2	3.76 ± 1.96	**0.000**^d^, p2	4.64 ± 2.58	**0.001**^d^ p2	0.181^a^
	T3	3.64 ± 2.80	0.237^d^, p3	4.48 ± 2.65	0.166^d^ p3	0.166^b^
	**p**	**0.000** c		**0.000** c		
**ODI**	T0	37.74 ± 13.76		47.02 ± 18.63		0.050^a^
	T1	30.26 ± 15.70	**0.000**^d^, p1	41.24 ± 15.93	**0.000**^d^ p1	**0.017** a
	T2	25.95 ± 12.79	**0.002**^d^, p2	35.70 ± 18.56	**0.008**^d^ p2	0.108^b^
	T3	26.46 ± 15.15	0.616^d^, p3	34.21 ± 20.29	**0.025**^d^ p3	0.285^b^
	**p**	**0.000** c		**0.000** c		
**SF-36** Physical Functioning	T0	49.00 ± 16.52		45.45 ± 21.87		0.530^a^
	T1	60.80 ± 21.54	**0.000**^d^, p1	50.45 ± 19.57	**0.037**^d^, p1	0.094^a^
	T2	68.60 ± 17.53	**0.001**^d^, p2	56.36 ± 21.34	**0.012**^d^, p2	**0.036** a
	T3	68.40 ± 18.18	0.515^d^, p3	56.14 ± 23.04	0.966^d^, p3	**0.047** a
	**p**	**0.000** c		**0.000** c		
Physical Role Limitation	T0	20.00 ± 28.87		18.18 ± 25.80		0.906^b^
	T1	25.00 ± 34.61	0.312^d^, p1	21.59 ± 24.76	0.445^d^, p1	0.889^b^
	T2	54.00 ± 36.57	**0.000**^d^, p2	40.91 ± 41.94	**0.013**^d^, p2	0.258^a^
	T3	63.00 ± 38.94	**0.025**^d^, p3	44.32 ± 42.21	0.083^d^, p3	0.121^a^
	**p**	**0.000** c		**0.000** c		
Emotional Role Limitation	T0	49.32 ± 42.08		28.80 ± 38.90		0.090^a^
	T1	56.00 ± 43.81	0.288^d^, p1	39.01 ± 43.30	**0.014**^d^, p1	0.189^a^
	T2	76.00 ± 37.91	**0.019**^d^, p2	52.27 ± 44.34	0.054^d^, p2	0.054^a^
	T3	81.34 ± 37.37	0.083^d^, p3	54.92 ± 46.77	0.306^d^, p3	**0.036** b
	**p**	**0.000** c		**0.000** c		
Energy/Fatigue	T0	49.60 ± 17.32		44.09 ± 16.01		0.265^a^
	T1	51.40 ± 17.88	0.639^d^, p1	44.32 ± 14.17	0.777^d^, p1	0.143^a^
	T2	53.20 ± 18.48	0.228^d^, p2	45.23 ± 16.29	0.816^d^, p2	0.125^a^
	T3	57.00 ± 18.48	**0.035**^d^, p3	45.91 ± 13.77	0.638^d^, p3	**0.025** a
	**p**	**0.005** c		0.974 c		
Emotional well-being	T0	65.28 ± 17.62		58.91 ± 16.62		0.210^a^
	T1	66.24 ± 18.91	0.711^d^, p1	63.82 ± 14.53	**0.002**^d^, p1	0.628^a^
	T2	68.32 ± 16.45	0.123^d^, p2	67.27 ± 13.84	0.111^d^, p2	0.815^a^
	T3	71.84 ± 15.16	**0.030**^d^, p3	65.82 ± 11.95	0.404^d^, p3	0.141^a^
	**p**	**0.005** c		**0.001** c		
Social Functioning	T0	55.50 ± 25.54		48.34 ± 21.29		0.306^a^
	T1	56.00 ± 25.55	0.659^d^, p1	55.34 ± 22.28	**0.004**^d^, p1	0.925^a^
	T2	62.50 ± 23.66	**0.040**^d^, p2	59.52 ± 18.77	0.382^d^, p2	0.638^a^
	T3	65.00 ± 26.52	0.331^d^, p3	60.27 ± 20.32	0.576^d^, p3	0.500^a^
	**p**	**0.025** c		**0.003** c		
Pain	T0	29.50 ± 13.19		22.05 ± 13.49		0.062^a^
	T1	42.10 ± 18.10	**0.001**^d^, p1	30.23 ± 16.51	**0.002**^d^, p1	**0.023** a
	T2	54.40 ± 21.04	**0.002**^d^, p2	44.43 ± 18.52	**0.002**^d^, p2	0.093^a^
	T3	56.40 ± 25.69	0.107^d^, p3	46.48 ± 22.69	0.259^d^, p3	0.169^a^
	**p**	**0.000** c		**0.000** c		
General Health	T0	55.20 ± 18.96		42.27 ± 16.09		**0.016** a
	T1	60.20 ± 18.85	**0.009**^d^, p1	45.45 ± 16.25	**0.011**^d^, p1	**0.006** a
	T2	64.60 ± 17.50	**0.035**^d^, p2	50.91 ± 17.64	0.067^d^, p2	**0.010** a
	T3	63.60 ± 18.88	0.708^d^, p3	53.18 ± 15.40	0.306^d^, p3	**0.039** a
	**p**	**0.000** c		**0.006** c		

PFOI, Periforaminal ozone injection; TFESI, Transforaminal epidural steroid injection; LBP, Low back pain; RP, Radicular pain; VAS, Visual analogue scale; ODI, Oswestry disability index

SD, Standard deviation

T0, Before treatment; T1, 2^nd^ week; T2, 1^st^ month; T3, 2^nd^ month

p1 , T0–T1;

p2 , T1–T2;

p3 , T2–T3

a , T-test;

b , Mann-Whitney U test;

c , Friedman test;

d , Wilcoxon test
